# Mechanical and Microcrack Evolution Characteristics of Roof Rock of Coal Seam with Different Angle of Defects Based on Particle Flow Code

**DOI:** 10.3390/ma16041401

**Published:** 2023-02-07

**Authors:** Qinghai Deng, Jiaqi Liu, Junchao Wang, Xianzhou Lyu

**Affiliations:** 1 College of Earth Science and Engineering, Shandong University of Science and Technology, Qingdao 266590, China; 2 Longshou Mine of Jinchuan Group Co., Ltd., Jinchuan 737100, China

**Keywords:** rock mechanics, microcrack evolution, defects angles, failure modes, multi-scale characterization, particle flow code

## Abstract

The creation of the natural ceiling rock of the coal seam is rife with fractures, holes, and other flaws. The angle of the defects has a significant influence on the mechanical characteristics and crack evolution of coal seam roof rock. Multi-scale numerical simulation software PFC2D gets adapted to realize the crack propagation and coalescence process in the roof rock of a coal seam with different angles of defects under uniaxial compression. The effect of flaw angles on the micro and macro mechanical characteristics of rock is also discovered. The results show that: (1) the defect angle has influence on the stress-strain, elastic modulus, peak strength, peak strain, acoustic emission (AE) and strain energy of roof rock of coal seam. When the defect angles are different, the starting position of the roof rock in a coal seam fracture is different. The quantity of microcracks firstly reduces with an increase in defect angles before gradually increasing. At the same fault angle, the cracks are mostly tensile ones and only a few shear ones. (2) When the defect angle is less than 90°, tensile and shear fractures are mostly localized at the defect’s two tips and propagate along the loading direction. When the defect angle is 90°, the tensile and shear cracks are not concentrated at the tip of the defect. (3) As the defect angles increase, the elastic strain energy rises initially and then falls, and the dissipated energy and total input energy both increase continuously. The elastic strain energy is greatest at the highest strength. The study provides a certain reference for the use of various analysis methods in practical engineering to evaluate the safety and stability of rock samples with pre-existing defects.

## 1. Introduction

In mining engineering, defective rock mass is a type of complicated engineering medium, as is water conservancy engineering, transportation engineering, and underground tunnel engineering [1,2,3]. The complex structure of rock makes its physical properties inhomogeneous and anisotropic, which makes the research of rock masses with defects more difficult [4,5,6,7]. In previous studies on fractured rocks, cracks are mostly placed in the middle of rock specimens [8,9,10,11,12]. When the rock specimen’s top or lower half is fractured, what effects will the angle change have on its mechanics and crack evolution characteristics.

At present, the research work on rocks with defects is mainly based on indoor rocks and includes the material model test or numerical simulation test. Through the laboratory test of rock with defects, we can find some influence rules such as inclination angle and number of prefabricated cracks, which have effect on the axial compressive strength, crack initiation, propagation and failure mode. And the numerical simulation software can only change one factor of the defect when other conditions remain unchanged, so that the research on the mechanical characteristics of defective rocks can be validated [13,14,15,16,17]. By controlling the numerical software program, the stress condition and changes of cracks near the defect can be obtained, which cannot be obtained experimentally. Hence, further analysis of the crack model’s stress variation properties during uniaxial compression can be done. For example, Yang et al. used PFC2D to study the tensile strength and fracture propagation of intermittently double-fractured rocks and discovered the effect of cracks on these properties. [18]. Su et al. conducted laboratory experiments to investigate how longitudinal fractures affect the mechanical characteristics of sandstone and obtained some useful research results [2]. Through indoor experiments, Yang et al. studied the law and mechanism of fracture propagation for various inclination defects. The test results have important guidance and reference significance for underground engineering construction design. [19]. Cao et al. have done a lot of research on fractured rock with pores, including laboratory tests, numerical simulation, and different influencing factors [20,21]. Li et al. investigated marble with holes’ dynamic failure properties under impact stress. The complete process of crack germination, propagation, penetration, and destruction was captured using a high-speed camera. [22]. The prefabricated hole specimen’s dynamic compressive strength, mechanism of failure, and crack propagation characteristics under impact stress were also explored.

It can be found that there are many kinds of research methods for rocks with cracks, cavities, and other defects. There are various research methods, including the indoor rock mechanics test, the numerical simulation test, the uniaxial compression test, triaxial compression test, the impact dynamic load test, and also the cyclic loading and unloading test, and so on. Hence, it is possible to obtain the rock’s mechanical, deformational, AE, failure, and energy properties, and so on. In addition, the effects of different crack combinations, water, heat, chemistry, and other environments on it are studied, and many useful results are obtained [23,24,25,26,27,28,29]. However, most of the defects in these studies are concentrated in the middle of the rock specimen or symmetrically distributed in the middle of the rock, and there are also a few studies on the distribution of shear cracks and tensile cracks in rock failure. However, in practical rock engineering, the distribution of cracks is not uniform. Hence, according to engineering practice, it is required to investigate the mechanical features of defects in a specific section. As fracture initiation and propagation are energy-driven processes, various analysis methods must be applied to assess the characteristics of rock samples with different defects.

In view of this, the numerical model of samples with various angle defects on one side of the roof of the coal seam is established using the PFC2D software, and its uniaxial compression is explored. The process of crack propagation and the number of microcracks are traced by using fish language [30,31,32]. Therefore, further analysis on the strain energy, strength, deformation, and acoustic emission with various crack inclinations is carried out, and the distribution characteristics of shear and tension cracks are also obtained.

## 2. Methodology

### 2.1. Particle Flow Code

**Figure 1:**
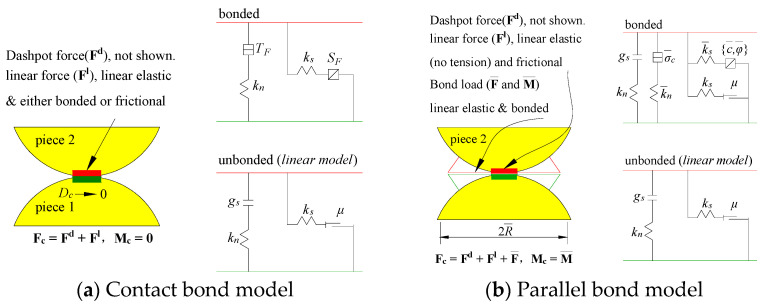
Bonded-particle model and its micro-mechanical behavior.

How particles interact with one another can be described by the particle contact constitutive model. Among them, the most commonly used are the contact bond model and the parallel bond model [30]. Infinitesimal, linear elastic, and interfacial bearing capacity characteristics can be provided by the contact bond model, whether the surface is bonded or frictional, as shown in Figure 1a. It can transmit force and moment at the same time. While the contact area of the contact bond model is point that can not transmit moment. The bond loses its function, and the spring will break, if the tensile or shear stress between the particles is greater than the normal or tangential bond strength. This means that the contact bond model is usually suitable for soil. While the parallel bond model can efficiently simulate the bonding between rock particles because it can regard the bonding between particles as a group of parallel springs with the functions of tension, shear, and torque, as shown in Figure 1b [31]. Hence, the parallel bond model is widely used in building PFC2D rock models and simulations [18,21,30].

### 2.2. Parameter Calibration of Roof Rock of Coal Seam

**Figure 2:**
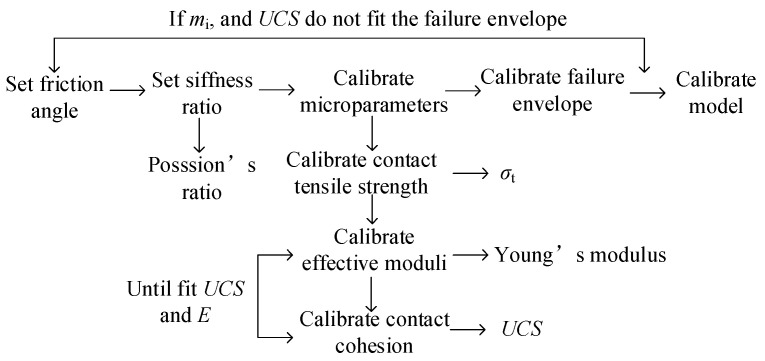
Parameter checking process of “trial and error” method for PFC model.

The purpose of meso-parameter calibration is to make the macro-mechanical properties of rock match the established PFC2D model. The meso-parameters include two parts: particle meso-parameters and meso-parameters of the contact constitutive model. The meso-parameters are usually calibrated by the “trial and error” method [18,21,30,31,32]. The most popular technique for calibrating the meso-parameters of the rock PFC2D model is “trial and error,” which involves gradually modifying the meso-parameters until the numerical test results are consistent with the rock’s large-scale mechanical properties. Figure 2 describes the parameter checking process of the “trial and error” method for the PFC model (version 5.0) [30,31,32].

The parameters provided by previous studies were used to conduct numerical tests because of the limitations of laboratory testing. Through the method of “trial and error” with repeated check comparisons, we obtained the physical mechanical characteristics for the PFC model, as shown in Table 1. Certainly, they were close to the macroscopic mechanical parameters of the real rock.

### 2.3. Numerical Models Roof Rock of Coal Seam with Different Defects Angle

**Figure 3:**
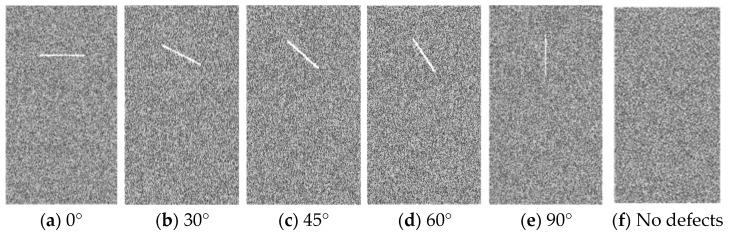
Numerical roof rock of coal seam specimens with different angle of defect and no defects.

Five rock models with varying angles of defects and one model without defects were built to explore the mechanical properties and the crack evolution law (Figure 3). The values in Table 1 were used to create these models as intact rocks first, and then defects were deleted before the simulation run. A certain thickness of grain element is deleted to simulate fracture. This is the most commonly used method at present and is mainly used to simulate discontinuous and unclosed fractures [18,21,30]. This is because after deleting the particles, the particles on both sides have been separated without any interaction. The loading rate needs to be low enough to guarantee the quasistatic loading condition. Hence, the loading rate in this study is set at 0.05 mm/s.

## 3. Numerical Simulation and Results

### 3.1. Strength and Deformation Properties

**Figure 4:**
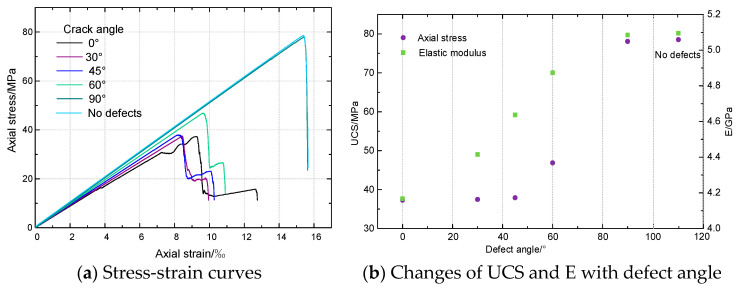
Stress-strain curves, UCS and E of rock with different defect angle.

Uniaxial compressive strength (UCS), elastic modulus (E), and stress-strain curves of rock samples with various defect angles are shown in Figure 4a,b. The defect angle has influence on the stress-strain curve, UCS and E of rock. Three stages, referred to as the elastic stage, plastic stage, and failure stage, may be distinguished between the stress-strain of rocks with various defect angles. Although the PFC rock model’s particles are rigid and have no initial damage, the numerical rock model’s stress-strain curve does not have a closed stage at the initial elastic stage similar to that of real rock. That is due to the fact that the PFC model is relatively homogeneous, which makes it beneficial to study the influence of defects from different angles on rock mechanical properties.

UCS increases with the increase of angle, but the increase is different with different angles. When the crack angle is below 45°, the UCS increases from 37.30 MPa at defect angle 0° to 37.91 MPa at defect angle 45° by 1.6%, then increases from 37.91 MPa at defect angle 30° to 78.1 MPa at defect angle 90° by 106%. Similarly, E has a similar trend, while its strain at maximal strength reduces initially, then increases as the angle increases.

**Figure 5:**
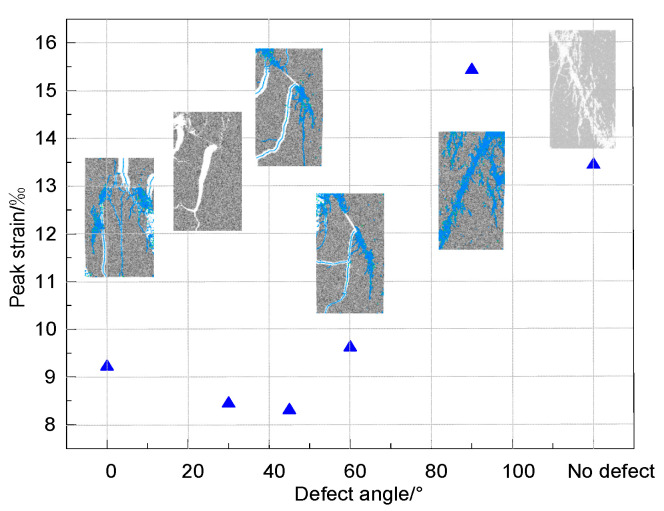
Peak strain of rock with different defect angle.

Figure 5 shows the peak strain and failure forms of rock specimens with different defect angles. It shows that when the angle rises, the peak strain initially reduces and then increases. The maximum is reached when the angle is 90°, and the value is 15.43‰. This is 85.68% higher than the minimum value (8.31‰) when the angle is 45°, and the peak strain value of rock without defect is 13.45‰.

### 3.2. Laws of Crack Evolution

**Figure 6:**
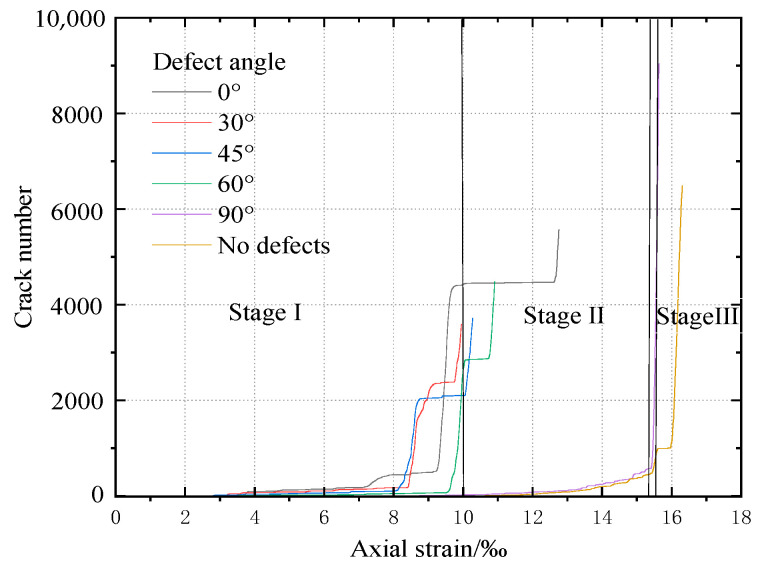
Number of cracks with axial strain of rock with different angle of defects.

Figure 6 shows the relationship between the axial strain and the number of cracks in rock samples with various defect angles. Three stages can be distinguished in the fracture development under uniaxial compression. Taking the relationship between the rock crack number and strain at an angle of 90° as an example, with continuous loading, the number of cracks can be divided into three stages: I, II, and III; the zero crack stage, the crack slowly propagating stage, and the crack rapidly growing stage, respectively. In addition, the number of cracks in rock without defects can also be divided into three stages with loading, but it is completely different from the rock with cracks. However, the evolution of microcracks in rock with defects has a stage of basically unchanged microcracks after the rapid increase of microcracks in the third stage, and then continues to appear the stage of rapid increase of microcracks.

**Figure 7:**
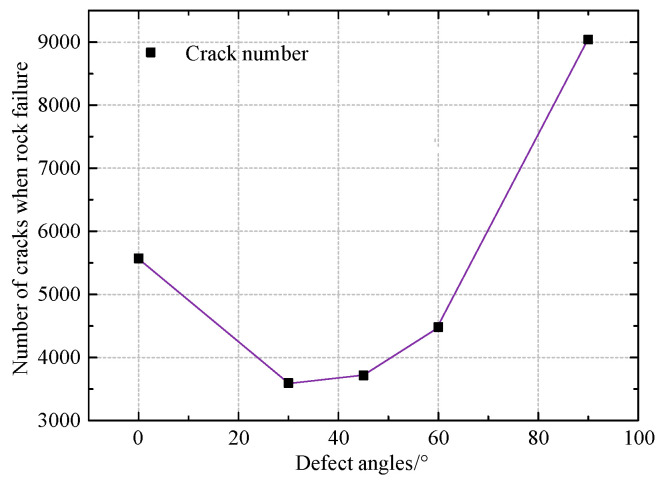
Number of cracks with different defect angles when rock failure.

To better understand the impact of defect angles and the development of the micro crack of rock samples, we list the number of microcracks when rock failure occurs with varied defect angles in Figure 7. It shows that the number of microcracks during rock failure initially decreases and subsequently increases as the defect angle rises. The relationship between the crack numbers and defect angles can be described by a nonlinear relationship, where the crack number during rock failure decreases from 5571 in defect angle 0° to 3594 in defect angle 30° by 35.5%, then increases from 3594 in defect angle 30° to 9040 in defect angle 90° by 151.5%.

### 3.3. Acoustic Emission Characteristics

As we all know, each crack in the PFC2D BPM creates an AE pulse. When the defect angle in rock samples is different, the AE characteristics under load are also different. And the AE event calculation of rock sample failure can be simulated by counting the number of cracks during uniaxial compression.

**Figure 8:**
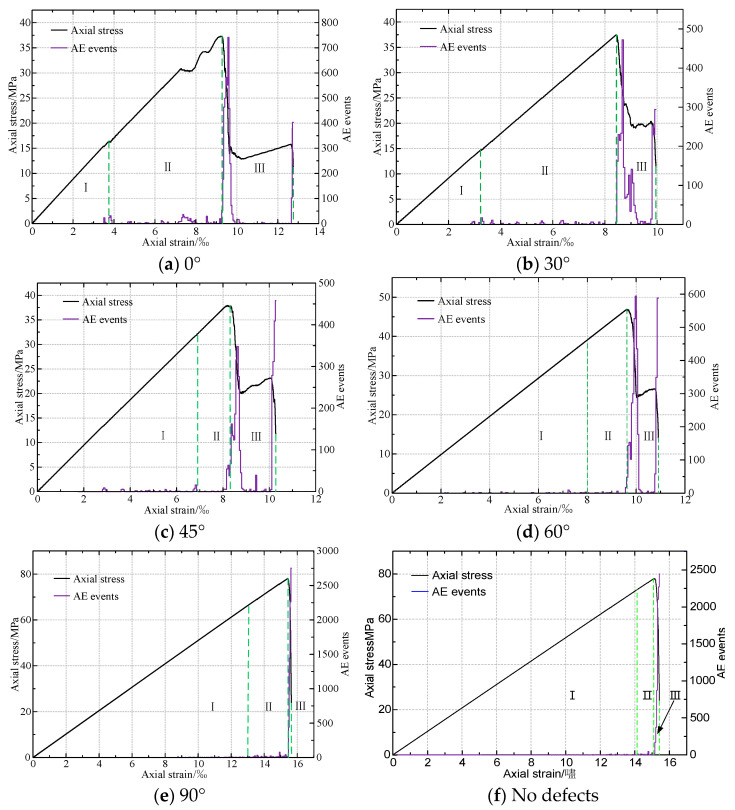
Stress-strain-AE events characteristics of rock with different angle of crack.

The stress-strain-AE event curves for various defect angles in rocks are shown in Figure 8. It can be seen that the AE event characteristics are strongly connected to the stress-strain relationship. The variation of AE events with strain can also be divided into three stages. In elastic stage, AE events rarely appear. The frequency of AE events rises when the stress-strain developed into plastic stage. When there is a failure, the AE events reach a peak and then rapidly decrease. It shows that the rock with different angles of cracking has been greatly damaged at this stage.

To sum up, the number of rock cracks significantly affects the number of AE events. The maximum AE events. The maximum AE events first decrease and then increase with the increase of the angle. The minimum value is 458 when the angle is 45°, and it is 2753 when the angle is 90°. In addition, when the angle is less than 90°, the AE events in the third stage are significantly different. When the angle is less than 90°, the third stage is relatively long, and there is a second peak of AE events. The AE events of rocks without defects are roughly similar to those of rocks with 90° defects, and there are basically no large AE events before the peak strength.

### 3.4. Crack Initiation and Distribution

**Figure 9:**
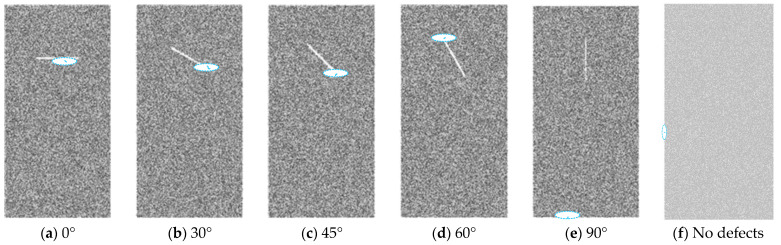
Crack initiation of rocks with defects at different angles.

In the process of simulation, the microcracks caused by intergranular bonding failure can be recognized as tensile microcracks and shear microcracks, which are represented by blue lines and green lines, respectively. The crack initiation of rocks with defects at different angles is shown in Figure 9. We can see that as the defect angles varied, the initial fracture position also varied. When the defect angle is 0°, the failure starts at the center, which is close to the lowest half of the defect. When the angle is 30, 45, and 60°, the fracture initiation occurs at the defect tip. The difference is that at 30° and 45°, the fracture starts at the lower part of the defect tip. When the angle is 60°, the fracture starts at the upper part of the defect tip. When the angle is 90°, the rock failure appears at the lower end of the whole rock, far away from the defect instead of appearing near the defect.

**Figure 10:**
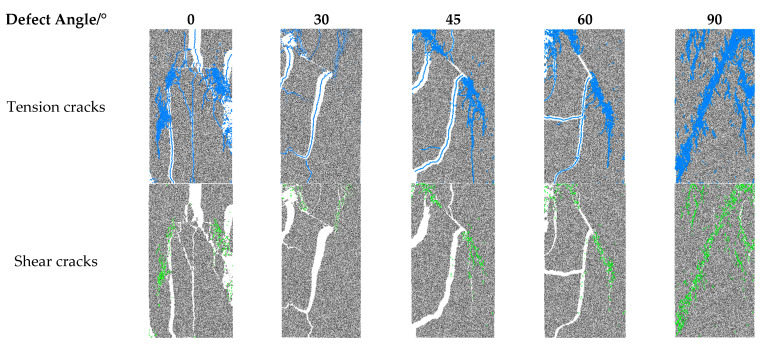
Distribution of tensile and shear cracks when rock failure.

Figure 10 shows the distribution of tension and shear cracks in samples with defects at various angles. It shows that at the same defect angle, the cracks of rock specimens are mainly tensile cracks, while shear cracks are relatively few. At different defect angles, tensile and shear crack counts initially drop and then subsequently rise. When the defect angle is 30°, the number of tensile cracks and reducing cracks is the least. We can also see that when the angle of the defect is less than 90°, the tensile crack and shear crack are mainly concentrated at the two tips of the defect and propagate along the loading direction. While the defect angle is 90°, the tensile and shear cracks are not concentrated at the defect tip. However, similar to the rock without defects, the microcracks are mainly concentrated on a fracture surface at about 45° in the horizontal direction. In Figure 4, the UCS and E of the 90° defect are much larger, which shows that they have little effect on the mechanical characteristics of rock. Accordingly, there are noticeably more tensile cracks than shear cracks when the defect angle is 90°.

### 3.5. Strain Energy Evolution Characteristics

**Figure 11:**
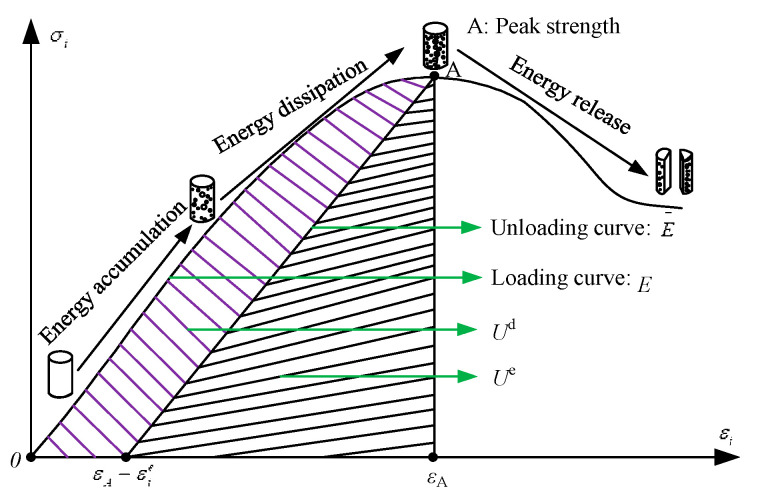
Relationship between dissipated energy and releasable strain energy in rock.

According to previous studies, rock failure is driven by energy release. Hence, a more accurate reflection of the rock failure law can be achieved if we can thoroughly understand the energy transfer and transformation during the process of rock loading until failure [33,34]. The stress-strain curve of a rock mass element is given in Figure 11. The area *U*^d^ represents the energy consumed by the element when damage and plastic deformation occur. The releasable strain energy kept in the cell is represented by the darkened region *U*^e^. $\overset{-}{E}$ is the unloading elastic modulus [34].

We assume that a unit volume rock mass element generates deformation when external force loading without experiencing any heat exchange with the environment. *U* is the total input energy that is produced by the external force work. According to the first law of thermodynamics [33,34]:

(1) $$U=U^{d}+U^{e}$$

where, *U^d^* is the dissipative energy, while *U^e^* is the elastic strain energy.

(2) $$U=\int \sigma_{1}d\varepsilon_{1}=\sum_{i=1}^{n}\frac{1}{2}\left(\sigma_{1i}+\sigma_{1i-1}\right)\left(\varepsilon_{1i}+\varepsilon_{1i-1}\right)$$

(3) $$U^{e}=\frac{1}{2\bar{E}}\left[\sigma_{1}^{2}+\sigma_{2}^{2}+\sigma_{3}^{2}-2\bar{\nu }\left(\sigma_{1}\sigma_{2}+\sigma_{2}\sigma_{3}+\sigma_{1}\sigma_{3}\right)\right]$$

where, $\overset{-}{\nu }$ and $\overset{-}{E}$ represent the mean values of the Poisson’s ratio and the unloading elastic modulus respectively.

The mathematical method used to determine the elastic strain energy that generate form triaxial compression is Equation (3). When the rock is under uniaxial compression ($\sigma_{2}=\sigma_{3}=0$), Equation (3) becomes:

(4) $$U^{e}=\frac{\sigma_{1}^{2}}{2\bar{E}}$$

**Figure 12:**
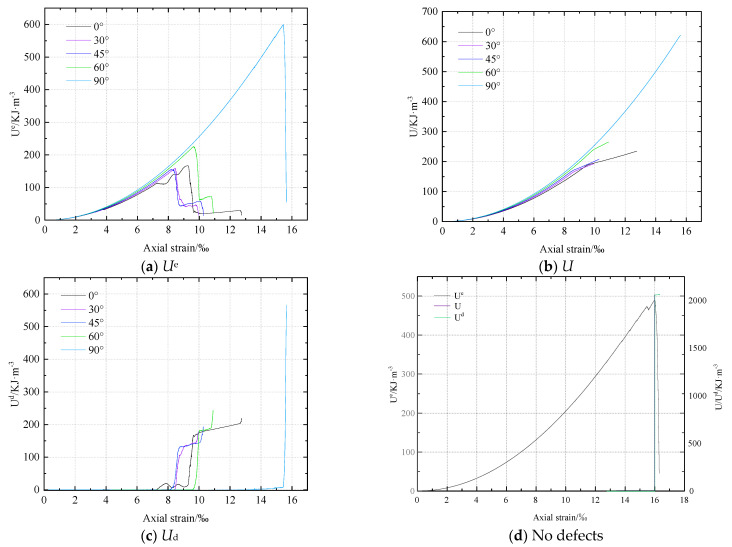
Variation diagram of total input energy *U*, elastic strain energy *U*^e^ and dissipative energy *U*^d^ of rock with strain at different defect angles and no defect.

The change trend of the total input energy *U*, elastic strain energy *U*^e^, and dissipative energy *U*^d^ of rocks with different defect angles is shown in Figure 12. Figure 12a shows that the elastic strain energy increases first and subsequently decreases with loading, reaching its maximum value when the rock is destroyed. With increasing loading, the total input energy tends to rise. At the end of the test, the total input energy can reach the maximum value. However, the dissipated energy does not change at first and then increases rapidly with the loading.

**Figure 13:**
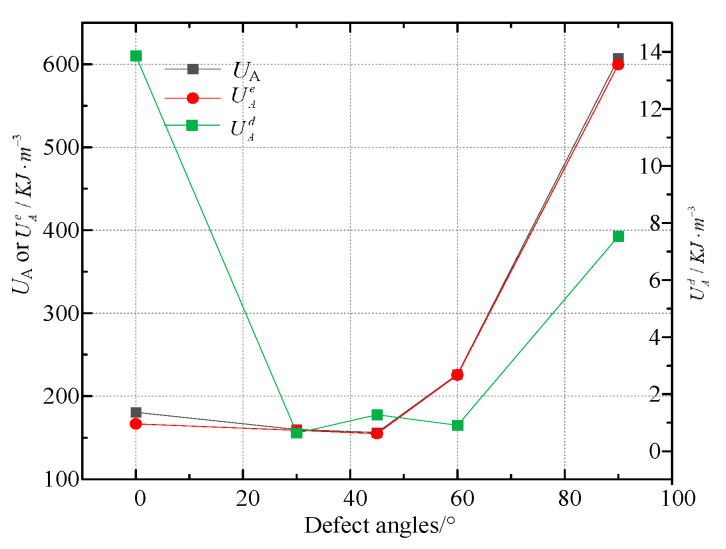
*U*_A_, $U_{A}^{e}$, and $U_{A}^{d}$ at the peak strength with different defect angles.

At the same time, we also calculated the total input energy *U*_A_, elastic strain energy $U_{A}^{e}$, dissipative energy $U_{A}^{d}$ and the proportion of $U_{A}^{e}$ and $U_{A}^{d}$ to *U*_A_ at the rock’s maximal strength with various defect angles is shown in Table 2 and Figure 13.

Table 2 and Figure 13 both show that when defect angles rise, *U*_A_ at the peak strength initially reduces and then subsequently increases. The minimum value is 156.23 KJ·m^−3^ at 45° and the maximum value is 607.04 KJ·m^−3^ at 90°. The difference between the minimum value and the maximum value is 74.3%. The value of *U*_A_ at peak strength also drops and subsequently increases with the increase in defect angles. The minimum value is 154.95 KJ·m^−3^ at 45°, and the maximum value is 599.50 KJ·m^−3^ at 90°. The difference between the minimum value and the maximum value is 74.2%. However, the dissipation energy $U_{A}^{d}$ at the peak strength is unstable with the increase of the defect angles. The minimum value is 0.64 KJ·m^−3^ at 30° and the maximum value is 13.87 KJ·m^−3^ at 0°, which have a 99.5% difference. In addition, it can be seen from Table 2 that the elastic strain energy at the peak strength of rock without defects is relatively small, while the dissipated energy is relatively large.

## 4. Conclusions

(1) The stress-strain curve, UCS, and *E* of rock are all significantly affected by the defect angle. The values of UCS become larger as the angle increases; however, the increase varies depending on the angle. *E* also presents such a trend. When the angle values increase, the strain values at peak strength drop first and then increase.

(2) The number of microcracks decreases first and then increases as the defect angle increases. The crack number when the roof rock of a coal seam failure decreases from 5571 at defect angle 0° to 3594 at defect angle 30° by 35.5%, then increases from 3594 at defect angle 30° to 9040 at defect angle 90° by 151.5%. With the increase of the defect angle, the maximum AE events first decrease and then increase. When the angle is less than 90°, the AE events in the third stage are significantly different. And when the angle is less than 90°, the third stage is relatively long, and there is a second peak of AE events.

(3) The starting position of rock failure is different at different angles of defects. When the defect angle is 0°, the failure starts in the middle of the defect, close to its bottom section. When the angle is 30, 45, or 60°, the fracture initiation occurs at the defect tip. When the defect is 90°, the rock failure occurs at the lower end of the whole rock far away from the defect, while not near the defect. Tensile fractures predominate at the same defect angle, but shear cracks are few.

(4) During loading, the elastic strain energy increases first and then decreases, reaching its peak value when the rock is broken. $U_{A}^{d}$ does not change at first and then increases rapidly with the loading. At the peak strength stage, *U*_A_ initially decreases and then increases. $U_{A}^{e}$ at the peak strength stage also decreases first and then increases. However, the dissipation energy at the peak strength is unstable with the increase of defect angles.

## Figures and Tables

**Table 1:** Micro mechanical parameters of coal and rock for numerical simulation.

Contact Parameter	Rock	Meaning
*R*_min_	0.2	Minimum particle size
*R*_max_/*R*_min_	1.5	Ratio of maximum particle size to minimum particle size
*E*_c_ (GPa)	1.8	Effective modulus of particles
*K*_n_/*K*_s_	1.5	Ratio of the contact stiffness between the normal direction and the tangential bond of particles
$\overset{-}{E}$ (GPa)	2.4	Bond effective modulus
$\overset{-}{K_{n}}/\overset{-}{K_{s}}$	1.5	Ratio of normal to tangential bonding contact stiffness
$\sigma_{b}$ (MPa)	16	Average and standard deviation of normal bond strength
$c_{b}$ (MPa)	20	Mean and standard deviation of cohesive force
ϕ (°)	42	Bond internal friction angle
$\overset{-}{\mu }$	0.5	Linear friction coefficient of particles

**Table 2:** *U*_A_, $U_{A}^{e}$, $U_{A}^{d}$ and the proportion at the peak strength of rock with different defect angles and no defects.

Defect Angles/°	*U*_A_/KJ·m^−3^	$U_{A}^{e}/\mathrm{KJ}\cdot m^{-3}$	$U_{A}^{d}/\mathrm{KJ}\cdot m^{-3}$	$U_{A}^{e}/U_{A}/\%$	$U_{A}^{d}/U_{A}/\%$
0	180.47	166.60	13.87	92.3	7.7
30	159.70	159.06	0.64	99.6	0.4
45	156.23	154.95	1.28	99.2	0.8
60	226.44	225.53	0.91	99.6	0.4
90	607.04	599.50	7.54	98.8	1.2
No defects	583.50	489.68	93.82	83.9	16.1

## Data Availability

The data used to support of this study are included within the article.
